# Supplementary material for: Changes of collagen content in lung tissues of plateau yak and its mechanism of adaptation to hypoxia
Source: PeerJ. 2024 Oct 1;12:e18250. doi: 10.7717/peerj.18250 (PMC11451445; doi:10.7717/peerj.18250)
Supplement: Supplemental Information 1 [file peerj-12-18250-s001.docx]

| Supplementary Material 1 Relative gene expression (log_10_N) N indicates absolute gene expression | | | | | | | | | | | | |
| --- | --- | --- | --- | --- | --- | --- | --- | --- | --- | --- | --- | --- |
| Mesenchymal collagen synthesis-related genes | COL1A1 | COL1A2 | COL2A1 | COL3A1 | COL9A2 | COL12A1 | COL14A1 | COL16A1 | COL21A1 | COL23A1 | COL26A1 | COL28A1 |
| XH-C | 1.631 | 1.717 | -0.832 | 2.154 | -0.754 | 1.773 | 0.853 | 0.744 | 0.864 | -0.124 | -0.209 | -0.399 |
| XH-Y | 2.370 | 2.532 | 0.065 | 2.683 | -0.364 | 1.899 | 1.492 | 1.307 | 1.559 | 0.553 | 0.566 | -1.416 |
| QML-Y | 1.524 | 1.788 | -3.000 | 1.896 | -3.000 | 1.545 | 1.341 | 1.004 | 1.276 | 0.452 | -0.029 | -1.553 |
|  |  |  |  |  |  |  |  |  |  |  |  |  |
| Basement membrane collagen synthesis-related genes | COL4A1 | COL4A2 | COL4A4 | COL4A5 | COL4A6 | COL8A1 | COL18A1 |  |  |  |  |  |
| XH-C | 2.522 | 2.367 | 1.186 | 1.209 | 0.211 | 1.579 | 1.503 |  |  |  |  |  |
| XH-Y | 2.810 | 2.610 | 1.436 | 1.315 | 0.607 | 1.662 | 1.620 |  |  |  |  |  |
| QML-Y | 2.261 | 2.145 | 1.021 | 0.806 | 0.388 | 1.310 | 1.388 |  |  |  |  |  |
|  |  |  |  |  |  |  |  |  |  |  |  |  |
| Peripheral collagen synthesis-related genes | COL5A1 | COL5A2 | COL5A3 | COL6A1 | COL6A2 | COL6A3 | COL6A5 | COL15A1 |  |  |  |  |
| XH-C | 1.236 | 1.632 | 0.611 | 2.002 | 2.148 | 1.459 | 0.852 | 1.395 |  |  |  |  |
| XH-Y | 1.725 | 1.679 | 0.780 | 2.386 | 2.560 | 1.947 | 1.185 | 1.582 |  |  |  |  |
| QML-Y | 1.373 | 1.348 | 0.408 | 2.145 | 2.319 | 1.503 | 1.307 | 1.363 |  |  |  |  |
